# L-Ferritin Binding to Scara5: A New Iron Traffic Pathway Potentially Implicated in Retinopathy

**DOI:** 10.1371/journal.pone.0106974

**Published:** 2014-09-26

**Authors:** Luísa Mendes-Jorge, David Ramos, Andreia Valença, Mariana López-Luppo, Virgínia Maria Rico Pires, Joana Catita, Victor Nacher, Marc Navarro, Ana Carretero, Alfonso Rodriguez-Baeza, Jesús Ruberte

**Affiliations:** 1 Interdisciplinary Centre of Research in Animal Health, Faculty of Veterinary Medicine, Universidade de Lisboa, Lisbon, Portugal; 2 Department of Morphology and Function, Faculty of Veterinary Medicine, Universidade de Lisboa, Lisbon, Portugal; 3 Center of Animal Biotechnology and Gene Therapy, Universitat Autònoma de Barcelona, Bellaterra, Spain; 4 Department of Animal Health and Anatomy, School of Veterinary Medicine, Universitat Autònoma de Barcelona, Bellaterra, Spain; 5 Department of Morphological Sciences, School of Medicine, Universitat Autònoma de Barcelona, Bellaterra, Spain; 6 CIBER de Diabetes y Enfermedades Metabólicas Asociadas (CIBERDEM), Barcelona, Spain; CINVESTAV-IPN, Mexico

## Abstract

Iron is essential in the retina because the heme-containing enzyme guanylate cyclase modulates phototransduction in rods and cones. Transferrin endocytosis is the classical pathway for obtaining iron from the blood circulation in the retina. However, the iron storage protein ferritin has been also recently proposed as an iron carrier. In this study, the presence of Scara5 and its binding to L-ferritin was investigated in the retina. Our results showed that Scara5, the specific receptor for L-ferritin, was expressed in mouse and human retinas in many cell types, including endothelial cells. Furthermore, we showed that intravenously injected ferritin crossed the blood retinal barrier through L-ferritin binding to Scara5 in endothelial cells. Thus, suggesting the existence of a new pathway for iron delivery and trafficking in the retina. In a murine model of photoreceptor degeneration, Scara5 was downregulated, pointing out this receptor as a potential player implicated in retinopathy and also as a possible therapeutic target.

## Introduction

Iron is required in cellular metabolism due to its participation in various heme and non-heme-containing enzymes [1]. The retina specifically needs iron because the enzyme guanylate cyclase assures the synthesis of cGMP, which acts as the second messenger in the phototransduction pathway [2]. Moreover, the extensive membrane biogenesis, necessary to continually replenish shed photoreceptor outer segments, also requires iron as an essential cofactor [3]. The retina obtains iron from the blood circulation. It is accepted that serum transferrin, the classical iron transporter protein, binds to its receptor on the surface of retinal pigment epithelial cells (RPE) and vascular endothelial cells and, in this way, free iron is delivered to the retina [4]–[6].

Involvement of iron in oxidative retinal damage has become clear [7]. The retina is constantly exposed to photo-oxidative stress, and it is especially vulnerable to damaging free radicals generated in the presence of the ferrous iron [8], [9]. Iron accumulation is associated with several retinopathies, including retinal degeneration [10], diabetic retinopathy [11], glaucoma [12], photoreceptor damage in uveitis [13], light-induced retinopathy [14], and age-related macular degeneration [15]. Despite its importance, iron influx and cell type involved in iron accumulation and storage mechanisms in the retina are not completely understood.

Ferritin is an iron handling protein ubiquitously distributed, known for its role in iron storage and detoxification [16]. Ferritin is composed of twenty-four subunits of heavy (H) and light (L) chains, whose ratio is variable in the different tissues [17]. The two chains of ferritin have complementary functions: H-ferritin possesses ferroxidase activity and enables the oxidation of iron; L-ferritin induces iron nucleation within the central core of the protein. Both ferritins can independently incorporate iron [18].

Recently, serum ferritin has been proposed as a new iron carrier protein [19]. Serum ferritin is composed mostly, but not exclusively, of L-ferritin [20], [21]. The advantage of serum ferritin as iron carrier, compared to serum transferrin, is that one molecule of transferrin only binds to two iron atoms, while serum ferritin can incorporate up to 4.500 iron atoms [19].

The blood-retinal barrier (BRB) prevents free access of blood-borne molecules to the retina [22]. Thus, specific receptors are required in order to allow serum ferritin influx into the retina. Ferritin specifically binds to several receptors: L-ferritin binds to Scara5 [23], while H-ferritin binds to TIM-2 [24], [25] and transferrin receptor 1 (TfR1) [26]. Nevertheless, neither Scara5 nor TIM-2 has so far been identified in the retina.

In this study, the presence of Scara5 and its binding to L-ferritin was investigated in the retina. Since iron transporters are frequently regulated by iron cytosolic levels, Scara5 expression was also evaluated in iron repletion conditions, and during degenerative retinopathy.

Our results showed that Scara5 is present in the mouse and human retina, and that serum ferritin can cross the BRB through its binding to Scara5 receptors. This pathway may constitute an important mechanism of iron traffic in the retina that is altered during experimental retinopathy.

## Material and Methods

### Ethics statement

Animal care and experimental procedures were approved by the Comissió d′Ètica en l′Experimentació Animal I Humana (CEEAH-Permit Number: 1666) of Universitat Autònoma de Barcelona (UAB). Mice were anesthetized with an intraperitoneal injection of a ketamine/xylazine mixture. Euthanasia was performed by inhalation with an overdose of isoflurane. For this study 45 mice were used.

Human eye samples were acquired from voluntary body donations to the Faculty of Medicine at UAB for teaching and research, in accordance with the Catalonian law (*DECRET 297/1997, de 25 de Novembre*). For this reason, it was not necessary specific ethics committee approval. Written informed consent was obtained from all adult participants. We received human eye samples without knowledge of the donor's identity, only biological data, such as sex and age, were available. Results obtained from the same human retinas were previously published [27], [28].

### Animals

Forty five CD1 adult mice were used. Animals were fed ad libitum with a standard diet (Panlab SL, Barcelona, Spain). Animal care and experimental procedures were approved by the Ethics Committee in Animal and Human Experimentation of the Autonomous University of Barcelona.

### Mouse retinopathy model experiments

Two groups of 6 mice each were intraperitoneally injected with 100 mg/kg of sodium iodate (NaIO3; Sigma-Aldrich, St Louis, MO, USA) in phosphate buffer and euthanatized 24 and 48 hours after treatment, as previously described [29]. A group injected only with physiological saline solution (PSS) was used as control. Retinas were obtained and processed according to the specific protocol for western blotting and immunohistochemistry.

### Ferritin intravenous administration experiments

Horse spleen ferritin (HSF; Sigma-Aldrich), 40 mg/mL, was injected in 8 animals. A group of four animals injected only with PSS was used as control. Six hours after HSF injection, retinas were obtained and processed according to the specific protocol for q-RT-PCR, western blotting and immunohistochemistry.

### Human retinas

Retinas from three healthy donors, two female and one man of 42 (D1), 86 (D3) and 78 (D2)-years-old, respectively, were formalin fixed and processed for immunohistochemistry.

### RNA isolation and cDNA preparation

Eyes were enucleated in an RNAse-free environment. Retinas were quickly and carefully dissected in sterile RNAse-free cold water solution, transferred and submerged immediately into 350 µl of RLT lysis buffer (Qiagen Inc, Hilden, Germany), homogenized with an Ultra-Turrax homogenizer (IKA-Labortechnik, Staufen, Germany). Total retinal RNA from each mouse was extracted using the RNeasy Mini Kit (Qiagen Inc) according to the manufacturer's protocol. To eliminate possible genomic DNA contamination, one column digestion of residual DNA was accomplished using an accessory RNase-free DNase set to use with RNeasy columns (Qiagen Inc), as the manufacturer's instructions. The total RNA was quantified by measuring the absorbance at 260 nm using a NanoDrop ND-2000c spectrophotometer (NanoDrop, Thermo Fisher Scientific, Willmington, DE, USA), and the purity was assessed by determining the ratio of the absorbance at 260 and 280 nm (NanoDrop), which indicated that all 260/280 nm ratios were>1.9. Integrity of the RNA was verified by visualization of the 28S and 18S ribosomal bands on 2% agarose gels containing ethidium bromide. For the RT-PCR, the High Capacity cDNA Reverse Transcription Kit (Applied Biosystems, Foster City, CA, USA) was utilized for synthesis of single stranded cDNA. Briefly, each RT reaction contained 700 ng of extracted total RNA sample using MultiScribe Reverse Transcriptase according to the manufacturer's instructions, with an RNase inhibitor in a final volume of 20 µl. The program is the following: 25°C 10 min, 37°C 120 min, 85°C 5 min. The cDNA aliquots obtained were stored at −20°C until further analysis.

### Quantitative real-time PCR analysis of gene expression

According to *Mus musculus* gene sequences, the primers were designed and when possible to across exon-exon boundaries using Primer3 (http://frodo/wi.mit.edu/primer3/) and Primer Express Software v. 2.0 (Applied Biosystems). The primer sequences, products lengths, and NCBI accession numbers tested are provided in Table 1. Primers were synthesized commercially by NZYTech (Lisbon, Portugal). Sequence homology searches were performed using the Basic Local Alignment Search Tool (Blast) “http://www.ncbi.nlm.nih.gov/blast”, to confirm gene identity of amplified fragments and that these primers matched only the sequence to which they were designed. To ensure optimal DNA polymerization efficiency, the amplicon length ranged between 95 and 165 bp. Before performing q-RT-PCR analysis, a final-point PCR was carried out for all genes to test the primers and verify the amplified products. PCR products were sequenced and homology searches were performed with Blast. Real time polymerase reaction was used to assess the retinal expression of scavenger receptor class A member 5 (putative) (*SCARA5*), transferrin receptor 1 (*TFRC*) and transferrin (*TRF*).

**Table 1:** Primer pairs sequences for quantitative q-RT- PCR.^1–3^

Gene symbol	Full gene name	Acc. Number 1	Primer pairs^3^ (5′-3′)	Exons spanned	Product size (bp)
***SCARA5***	scavenger receptor class A, member 5 (putative)	NM_001168318.1; NM_028903.2	F: agg agg gaa agc cag gta gc R: ccc cta gct tcc cat cat ca	5–6	108
***TFRC***	transferrin receptor	NM_011638.4	F: tgt gaa gct cat tgt gaa aaa cg R: gcg tct ctc tgg gct cct ac	10–11	117
***TRF***	transferrin	NM_133977.2	F: arc cga tgc tat gac ctt gg R: ccc ttc ttt acc aca gcc aca	3–4	144
***ACTB*** 2	actin, beta	NM_007393.3	F: tgt tac caa ctg gga cga ca R: ggg gtg ttg aag gtc tca aa	3–4	165
***GAPDH*** 2	glyceraldehyde-3-phosphate dehydrogenase	NM_008084.2	F: cgt gtt cct acc ccc aat gt R: gcc tgc ttc acc acc ttc tt	5–6	95

1 Entrez Gene, National Center for Biotechnology Information (NCBI).

2 housekeeping genes.

^3^ F: forward primer and R: reverse primer.

Gene relative quantification was carried out using MicroAmp Optical 96-well plates (Applied Biosystems) in a StepOnePlus thermocycler (Applied Biosystems) in standard cycling conditions. The 12.5 µl PCR reaction mixtures contained 6.25 µl of 2×Power SYBR Green PCR Master Mix (Applied Biosystems), 160 nM of each primer, and 2 µl of diluted cDNA as template. No transcription and no template samples were used as controls. The primer specificity and the formation of primer-dimers were confirmed by melt curve analysis and agarose gel electrophoresis. Measurements of each sample for each gene were made in duplicate and the relative quantification for each target gene was calculated using the normalization factor which was the geometric mean of beta-actin (ACTB)/glyceraldehyde-3-phosphate dehydrogenase (GAPDH) genes according to the 2^−ΔΔCt^ method [30]. Statistical analysis of relative expression values was performed using Rest 2009 V2.0.13 software (http://www.REST.de.com).

### Western blot analysis

For western blot analysis, animals were perfused with phosphate buffered saline solution (PBS) through the aorta to remove the traces of blood transferrin and serum ferritin. Eyes were enucleated, and retinas dissected and homogenized in modified RIPA buffer (25 mM Tris base, pH 8.2, 150 mM NaCl, 0.5% NP-40, 0.1% SDS, 0.5% sodium deoxycholate), containing protease inhibitor cocktail tablets (cOmplete Mini, Roche, Basel, Switzerland). Nuclear and cytoplasmic protein fraction samples were obtained using the ReadyPrep Protein Extraction Kit (Cytoplasmic/Nuclear) (Bio-Rad, Hercules, CA, USA) according to manufacturer's instructions. Livers were homogenized with an Ultra-Turrax (Ika-Labortechnik) in the lysis buffer described. Protein concentrations were determined with BCA protein assay reagent [31] and Bradford assay method [32]. For immunoblotting, lysates were resuspended in SDS–PAGE loading buffer (140 mM Tris base, pH 6.7, 6.8% SDS, 33% glycerol, 0.004% bromophenol), ultrasonicated for 10 min, heated at 95°C for 15 min, and centrifuged at 14000 rpm/8000 g for 10 min. 5% β-mercaptoethanol was added to the supernatants, followed by incubation on ice for 20 min. Prior to loading, protein samples were heated at 95°C for another 5 min, and were separated in a 12% pre-cast SDS-PAGE gel (Bio-Rad). Proteins were transferred to an Immobilon-P polyvinylidene fluoride (PDVF) membrane (Merck Millipore, Billerica, MA, USA) for antibody probing. The following primary antibodies were used: rabbit anti-mouse Horse Spleen Ferritin (Sigma-Aldrich) at 1∶2000 dilution, rabbit anti-mouse GFAP (DAKO, Glostruo, Denmark) at 1∶3000 dilution; rabbit anti-mouse L- ferritin (Abcam, Cambridge, UK) at 1∶2000 dilution; rabbit anti-mouse Scara5 (Abcam) at 1∶1000 dilution, rabbit anti-mouse Topoisomerase I (Abcam) at 1∶500 dilution; rabbit anti-mouse transferrin (Acris, Herford, Germany), at 1∶10000 dilution, and rabbit anti-mouse transferrin receptor 1 (Abcam) at 1∶1000. All primary antibodies were incubated in blocking buffer of 5% non-fat milk powder in PBT (Phosphate buffered saline, 0.05% Tween-20) for 2 hours at room temperature. After washing with PBT, membranes were incubated for 30 min at room temperature with a horseradish peroxidase-conjugated goat anti-rabbit IgG secondary antibody (Southern Biotech, Birmingham, AL, USA) at 1∶75000 dilution in blocking buffer. Following washing with PBT, detection was performed by enhanced chemiluminescence using Luminata Crescendo (Merck Millipore). A rabbit anti-mouse alpha-tubulin primary antibody (Abcam) at 1∶600000 was used to normalize blot loading.

### Immunohistochemistry

Eyes embedded in paraffin were sectioned (3 µm) along the eye axis through the optic disc and cornea, deparaffinized and rehydrated. Whole-mount retinas were fixed in 10% neutral buffered formalin for 2 hours at 4°C. After they were washed in PBS, paraffin-sections and whole mount retinas were incubated overnight at 4°C with the following antibodies: goat anti-mouse α-SMA (Abcam) at 1∶100 dilution; rat anti-mouse Brn3a (Santa Cruz Biotechnology, Inc, Heidelberg, Germany) at 1∶100 dilution; rat anti-mouse CD34 (Biolegend, San Diego, CA, USA) at 1∶50 dilution; goat anti-mouse collagen IV (Millipore, Temecula, CA, USA) at 1∶200 dilution; goat anti-mouse Iba1 (Abcam) at 1∶500 dilution; rabbit anti-mouse GFAP (DAKO) at 1∶1000 dilution; rabbit anti-mouse GS (Sigma-Aldrich) at 1∶100 dilution; rabbit anti-mouse Horse Spleen Ferritin (GeneTex, Irvine, CA, USA) at 1∶100 dilution; rabbit anti-L-ferritin (Abcam) at 1∶500 dilution; mouse anti-mouse PKC (Sigma-Aldrich) at 1∶500 dilution; rabbit anti-Scara5 (LSBio, Seattle, WA, USA) at 1∶200 dilution; rabbit anti-mouse transferrin (Abcam) at 1∶100 dilution; rabbit anti-mouse transferrin receptor 1 (Abcam) at 1∶100 dilution; rat anti-mouse 2F8 (Serotec, Oxford, UK) at 1∶100 dilution, and PNA Lectin (Sigma-Aldrich) at 1∶100 dilution. After they were washed in PBS, the retinas were incubated at 4°C overnight with specific secondary antibodies: biotinylated anti-goat (1∶100) and anti-rabbit (1∶100) IgGs (Vector Laboratories, Burlingame, CA, USA). After washing in PBS, streptavidin Alexa Fluor 488 and 546 conjugates were used as fluorochromes (Invitrogen, Carlsbad, CA, USA) at 1∶100 dilution; the incubation was made at 4°C overnight. Nuclear counterstaining with To-Pro-3 (Invitrogen), at 1∶100 dilution, or with Hoechst stain solution (Sigma-Aldrich) was performed for microscopic analysis with the laser scanning confocal microscope (TCS SP2; Leica Microsystems GmbH, Heidelberg, Germany). The 2F8 antibody was revealed with 3,3′-diaminobenzidine (DAB) and paraffin sections were counterstained with hematoxylin. Procedural immunohistochemistry controls were done by omission of the primary antibody in a sequential tissue section.

## Results

### Scara5 was expressed in mouse retina

To investigate the presence of Scara5 in mouse retinal cells, *SCARA5* mRNA levels and Scara5 protein expression were analyzed by means of q-RT-PCR and western blotting, respectively. Liver was used as a positive control.

**Figure 1:**
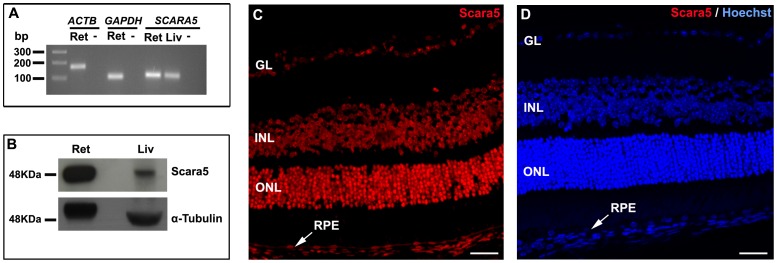
Scara5 was expressed in mouse retinal cells. A: The expression of *SCARA5* mRNA in the retina was evaluated by q-RT-PCR. Agarose gel electrophoresis of q-RT-PCR products confirmed that *SCARA5* single amplicon with 108 bp was generated. *ACTB* and *GAPDH* were used as housekeeping genes. B: Western blotting analysis revealed a specific band with a molecular weight of 48 KDa, confirming the presence of Scara5 receptors in the retina. α-tubulin was used as a loading control. C: Retinal immunolabeling with a specific antibody in a histological section, along the eye axis through the optic disc and cornea, showed that Scara5 was expressed throughout the retina, mainly at the level of ganglion cell layer, inner nuclear layer, outer nuclear layer and RPE. D: Immunohistochemical negative control, where the primary antibody was omitted. Ret, retina; Liv, liver; -, no-template control; GL, ganglion cell layer; INL, inner nuclear layer; ONL, outer nuclear layer; RPE, retinal pigment epithelium. Scale bars: 28 µm (A); 28 µm (B).

**Figure 2:**
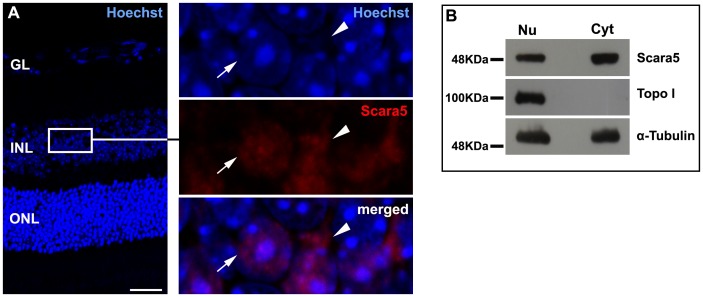
Scara5 was found both in the nucleus and cytoplasm of retinal cells. A: Scara5 expression was observed at nuclear (arrow) and cytoplasmic (arrowhead) compartments in mouse retinal histological sections immunolabeled with a specific anti-Scara5 antibody. Nuclei were marked with Hoechst Stain Solution. B: Western blotting analysis of nuclear and cytoplasmic protein fractions samples confirmed the nuclear and cytoplasmic Scara5 content in the retinal cells. α-tubulin was used as a loading control. Topoisomerase-I (Topo I) was used to assure that nuclear protein was not present in the cytoplasmic fraction. GL, ganglion cell layer; INL, inner nuclear layer; ONL, outer nuclear layer. Scale bar: 28 µm.

Our results revealed mRNA transcript and protein expression of Scara5 in the retina (Figure 1A and B). The immunolabeling of paraffin-embedded retinal sections with a specific anti-Scara5 antibody confirmed the expression of Scara5 throughout the neuroretinal parenchyma and RPE. A stronger immunoreactivity was observed at the ganglion cell layer, inner nuclear layer, outer nuclear layer, and photoreceptor inner segments (Figure 1C). In addition, Scara5 expression was detected both in the nucleus and cytoplasm of retinal cells (Figure 2A).

To rule out the possibility of nonspecific labeling of the antibody in the nucleus, nuclear and cytoplasmic protein fraction samples were analyzed by western blotting. Our results confirmed that Scara5 was expressed both at nuclear and cytoplasmic compartments in retinal cells (Figure 2B).

**Figure 3:**
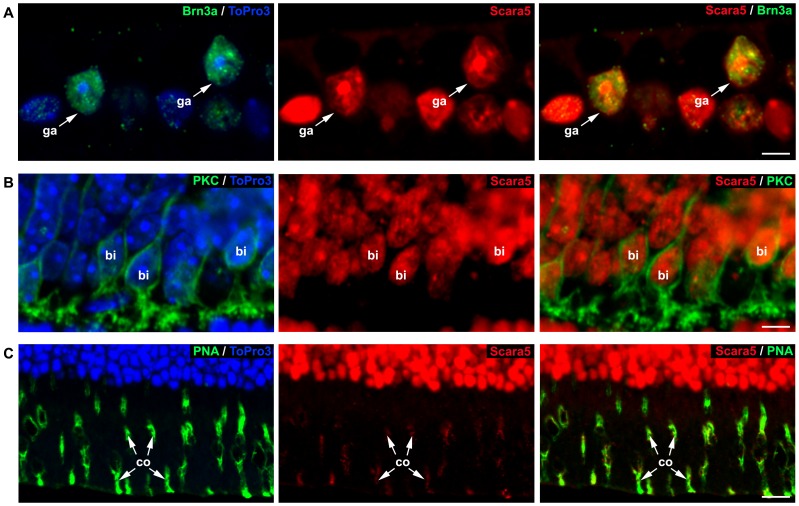
Scara5 was expressed in retinal neurons. A: Labeled retinal ganglion cells with Brn3a showed a nuclear intense Scara5 signal. B: PKC positive bipolar cells expressed Scara5. C: Photoreceptors presented a high expression of Scara5. The labeling with PNA lectin, which is specific for cone inner segments, showed a more intense signal in cones than in rods. Nuclei were counterstained with ToPro3. ga, retinal ganglion cells; bi, bipolar cells; co, cone inner segments. Scale bars: 3,8 µm (A); 5,4 µm (B); 12,5 µm (C).

**Figure 4:**
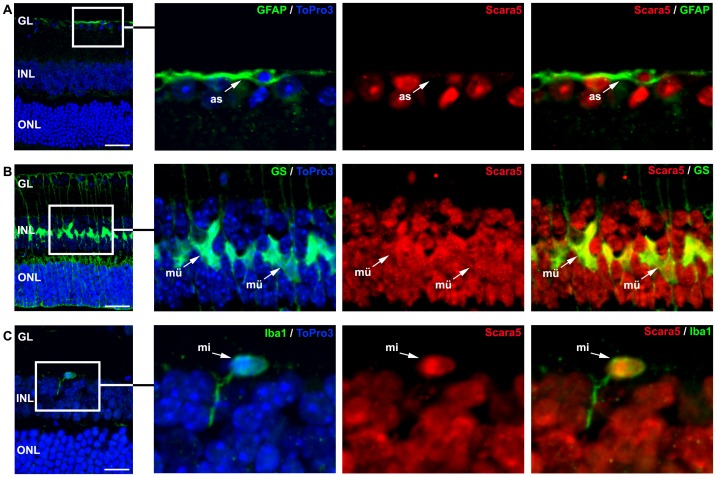
Scara5 was expressed in retinal glia. A: Labeled astrocytes with GFAP showed Scara5 expression. B: GS positive Müller cells presented intense Scara5 signal both at the nuclear and cytoplasmic compartments. C: Microglial cells revealed a more intense Scara5 signal in nucleus than in cytoplasm. Nuclei were counterstained with ToPro3. as, astrocyte; mü, Müller cell; mi, microglial cell. GL, ganglion cell layer; INL, inner nuclear layer; ONL, outer nuclear layer. Scale bars: 33 µm (A); 33 µm (B); 14,7 µm (C).

Dual staining with specific markers (Brn3a, PKC and PNA lectin) against retinal neurons [33], [34], [35] showed that ganglion cells, bipolar cells and photoreceptors expressed Scara5 (Figure 3). The content of Scara5 was higher in the inner segment of cones in comparison with rods (Figure 3C). Scara5 was also expressed in astrocytes, Müller cells and microglial cells, as shown by the co-localization of anti-Scara5 antibody with anti-GFAP, anti-GS and anti-Iba1 antibodies, respectively (Figure 4).

### L-ferritin expression pattern in the retina

Since L-ferritin is the preferred ligand of Scara5 [23], we explored L-ferritin expression and its spatial distribution along the retinal parenchyma by means of q-RT-PCR, western blotting and immunohistochemistry.

**Figure 5:**
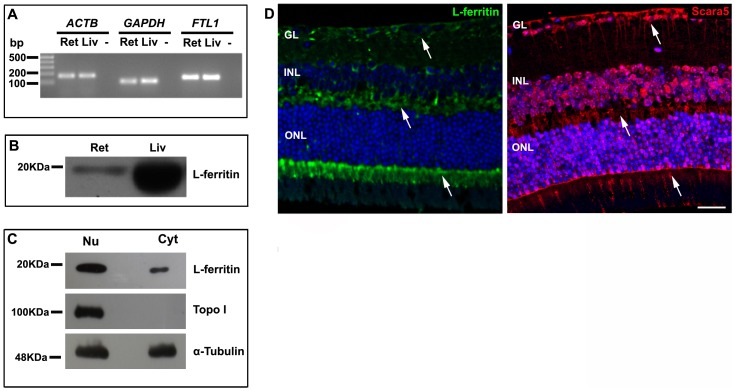
L-ferritin expression in mouse retina. A: The expression of *FTL1* mRNA in the retina was evaluated by q-RT-PCR. Agarose gel electrophoresis of q-RT-PCR products confirmed that *FTL1* single amplicon with 144 bp was generated. *ACTB* and *GAPDH* were used as housekeeping genes. B: Western blotting analysis showed a specific band with a molecular weight of 19 KDa, confirming the presence of L-ferritin in the retina. α-tubulin was used as a loading control. C: Analysis of nuclear and cytoplasmic protein fractions samples showed that L-ferritin was present in both cellular compartments. α-tubulin was used as a loading control. Topo I was used to assure that nuclear protein was not present in the cytoplasmic fraction sample. D: As expected, L-ferritin immunolabeling distribution pattern (arrows) was in accordance with the Scara5 signal (arrows) in paraffin-embedded retinal sections. Ret, retina; Liv, liver; -, no-template control; GL, ganglion cell layer; INL, inner nuclear layer; ONL, outer nuclear layer. Scale bar: 29 µm.

In agreement with previous studies [36], [37], our results showed *FTL1* mRNA transcript and protein expression in the retina (Figure 5A and B). Moreover, as occurred with Scara5, L-ferritin was found both in nuclear and cytoplasmic compartments (Figure 5C). Immunohistochemistry confirmed that L-ferritin was present throughout the retinal parenchyma, with a higher expression in the ganglion cell layer, outer plexiform layer, and photoreceptor inner segments. L-ferritin expression followed the distribution pattern of Scara5 (Figure 5D).

### Scara5 was detected in retinal vasculature

Since serum ferritin is mainly composed of L-ferritin [20], [21], we investigated the presence of Scara5 receptors in retinal blood vessels as a possible pathway for serum ferritin influx into the retina. The double immunostaining of paraffin-embedded mouse retinal sections against Scara5 and collagen IV, a widely used blood vessel basement membrane marker [38], Scara5 and CD34, that has a strong expression in retinal endothelial cells [39], and Scara5 and α-SMA, that in the retina is only found in vascular smooth muscle cells [40], was performed.

**Figure 6:**
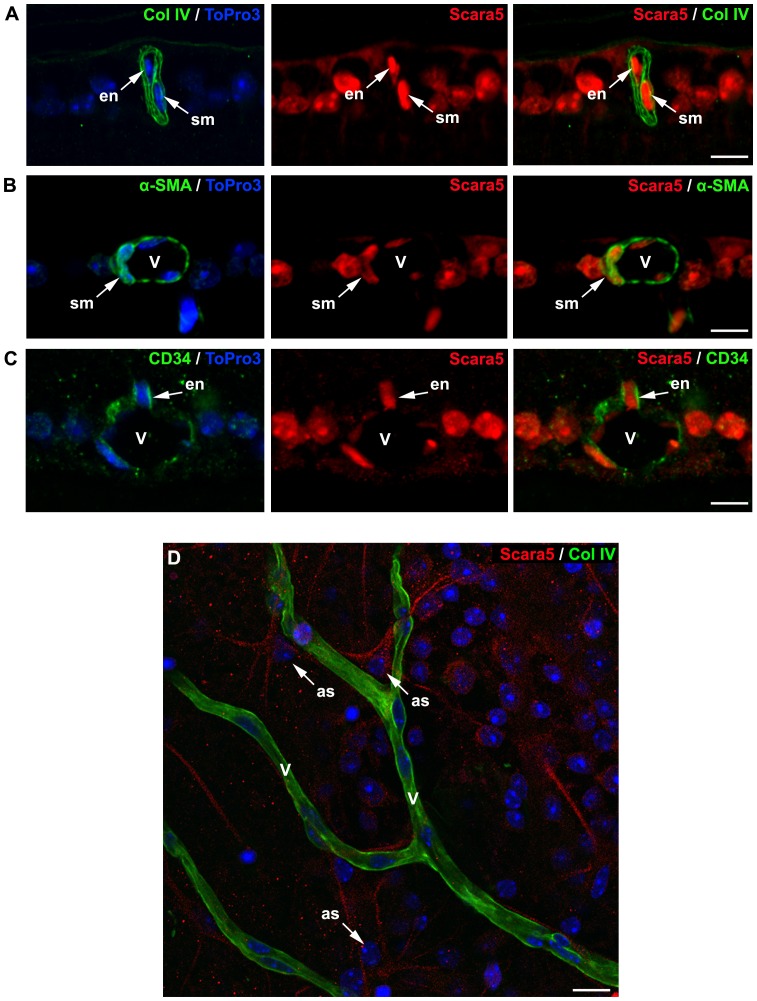
Retinal vasculature expressed Scara5 receptors. A: Cells surrounded by the blood basement membrane marked with collagen IV showed intense Scara5 signal. B and C: Dual immunolabeling with Scara5 and with α-SMA and CD34, respectively, confirmed that vascular smooth muscle cells and endothelial cells expressed Scara5 receptors. D: Whole mount retinas immunohistochemically marked with collagen IV and Scara5 showed that perivascular astrocyte-like cells intensively expressed Scara5 in their vascular end-feet. Nuclei were counterstained with ToPro3. en, endothelial cell; sm, smooth muscle cell; v, blood vessel; as: astrocyte-like cell. Scale bars: 10 µm (A); 9 µm (B); 9 µm (C); 12 µm (D).

Scara5 expression was detected in endothelial and smooth muscle cells of retinal vasculature (Figure 6), suggesting that serum ferritin could be transported across the BRB into the retinal parenchyma by L-ferritin binding to Scara5. Moreover, in whole mount retinas, astrocyte-like cells surrounding blood vessels also expressed Scara5 (Figure 6D).

### Serum ferritin influx into the retina

Due to the expression of Scara5 in endothelial cells, a possible Scara5 pathway for ferritin influx into the retina was explored. In order to test if serum ferritin crosses the BRB, mice were intravenously injected with 40 mg of HSF. L-ferritin is the main component of HSF [19]. Six hours after the injection, mice were euthanized, retinas obtained, and Scara5, L-ferritin, TfR1, and transferrin relative gene expression and protein levels were assessed with q-RT-PCR and western blotting.

**Figure 7:**
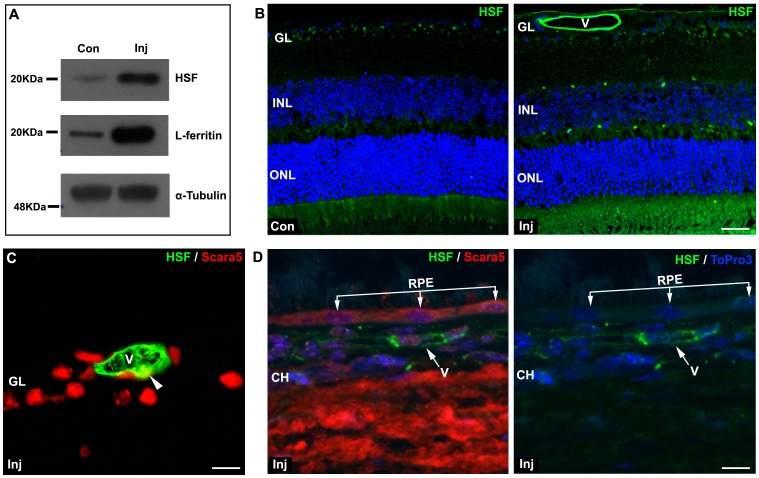
Intravenously injected HSF crossed the BRB. A: Six hours after the intravenous injection of HSF, western blotting analysis revealed that HSF was present in the retina. As expected, a marked increase of L-ferritin content was also confirmed. B: The immunolabeling with a specific anti-HSF antibody showed that HSF crossed the inner BRB and accumulated in mouse retina. HSF was internally lining the retinal blood vessels. C and D: The double staining with anti-HSF and with anti-Scara5 antibodies showed that L-ferritin co-localized with endothelial cytoplasmic Scara5 (arrowhead), but no content of HSF was observed in RPE cells, suggesting a differential function of the inner and outer component of BRB. Nuclei were counterstained with ToPro3. Con, non-injected control; Inj, injected; V, blood vessel; GL, ganglion cell layer; INL, inner nuclear layer; ONL, outer nuclear layer; RPE, retinal pigment epithelium; CH, choroid. Scale bars: 24 µm (B); 10 µm (C); 8 µm (D).

Our results showed that in healthy mice, without BRB breakdown, intravenously injected HSF was taken up and accumulated in the retina (Figure 7A and B). Interestingly, HSF was lining the internal surface of retinal vessels (Figure 7B). When Scara5 immunolabeling was performed in HSF injected retinas, colocalization of HSF and cytoplasmic Scara5 was observed in endothelial cells, suggesting that the binding between L-ferritin and Scara5 may serve as a mechanism for serum ferritin transport across the BRB (Figure 7C). The outer BRB barrier, the RPE, expressed Scara5 (Figures 1C and 7D), but surprisingly HSF from choroidal circulation was not detected in RPE cells, suggesting that L-ferritin did not cross the outer BRB (Figure 7D).

**Figure 8:**
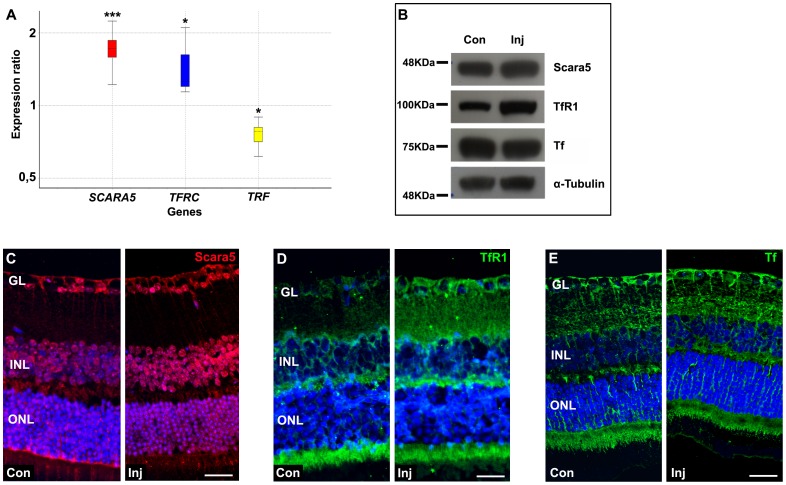
HSF influx into the retina changed Scara5, TfR1, and transferrin gene and protein expression. A: Relative expression of *SCARA5*, *TFRC*, and *TRF* obtained from q-RT-PCR analysis. Y-axis indicates the relative expression distribution of a ratio of injected mice retinas versus non-injected control mice retinas with *ACTB* and *GAPDH* as housekeeping genes. Boxes represent interquartile range, the median value is indicated by horizontal dotted line, and whiskers represent the minimum and maximum observation. Statistical significance calculated by Rest 2009 (http://www.REST.de.com) is indicated by *p<0,05, and ***p<0,001 (n = 12). Ratios over one indicate genes (*SCARA5* and *TFRC*) with higher expression in injected mice retinas relative to non-injected control mice retinas, and ratio less than one indicates gene (*TRF*) with lower expression in injected mice retinas opposed to non-injected control mice retinas. Western blotting and immunohistochemistry analyses confirmed that HSF accumulation in the retina was accompanied with an increased expression of Scara5 and TfR1 (B,C,D), and a slightly decrease of transferrin (B,E). Nuclei were counterstained with ToPro3. Con, non-injected control; Inj, injected; GL, ganglion cell layer; INL, inner nuclear layer; ONL, outer nuclear layer. Scale bars: 25 µm (C); 28 µm (D); 30 µm (E).

Iron handling proteins are usually regulated by cytosolic iron levels [7], [41]. The study of HSF injected mice retinas by q-RT-PCR revealed that *TRF* mRNA level was down-regulated ∼0.8-fold (P<0,037). In contrast, *SCARA5* and *TFRC* mRNA levels were up-regulated ∼1.7-fold (P<0,0001) and ∼1.4-fold (P<0,039), respectively (Figure 8A). Similar results were obtained by western blotting analyses (Figure 8B). The immunolabeling with specific antibodies for Scara5, TfR1, and transferrin confirmed this expression profile in HSF injected mice retinas (Figures 8C-E). Altogether, these findings suggested that HSF, composed by L- and H-ferritin, can cross the inner BRB through L-ferritin binding to Scara5 and H-ferritin binding to TfR1 in retinal blood vessels.

### Scara5 expression decreases during retinopathy

To investigate the possible involvement of Scara5 during retinopathy, we used a murine model of retinopathy with photoreceptor degeneration induced by the injection of sodium iodate [29]. The toxicity of sodium iodate induces sequential retinal structural and functional changes in a dose and time dependent manner [42], [43].

**Figure 9:**
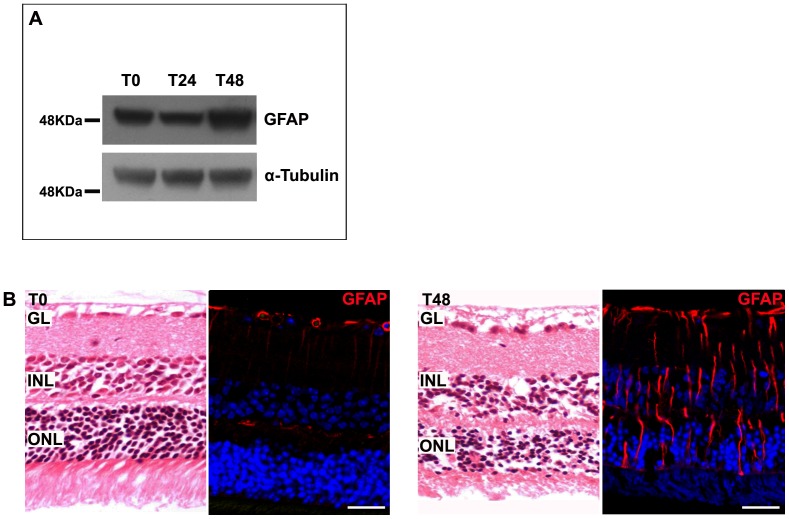
Murine model of retinopathy with photoreceptor degeneration. A: Forty-eight hours after sodium iodate injection, retinas analyzed by western blotting showed an increased expression of GFAP. α-tubulin was used as a loading control. B: Paraffin-embedded retinal sections stained with hematoxylin-eosin or immunolabeled with a specific anti-GFAP antibody revealed photoreceptor alterations and gliosis, indicating that retinopathy was well established. Nuclei were counterstained with ToPro3. GL, ganglion cell layer; INL, inner nuclear layer; ONL, outer nuclear layer. Scale bar: 35 µm.

Two groups of 6 mice each were intraperitoneally injected with 100 mg/kg of sodium iodate and euthanatized 24 and 48 hours after treatment. A group injected only with PSS was used as control. As expected, western blotting analysis and immunolabeling with anti-GFAP antibody, revealed an increased expression of GFAP (Figure 9A), and consequent gliosis (Figure 9B), indicating that, 48 hours after treatment, retinopathy was well established.

**Figure 10:**
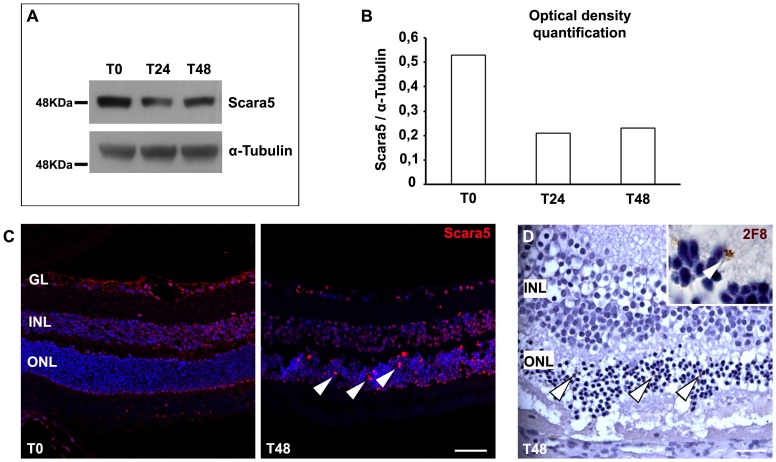
Scara5 expression decreased during retinopathy. A: Retinas of a sodium iodate murine model were analyzed by western blotting. Scara5 expression reduced to about half during the treatment. B: Graph representing the optical density quantification of the Western blotting analysis for Scara5, after normalization with respect to α-tubulin. C: The immunolabeling of paraffin-embedded retinal sections with anti-Scara5 antibody confirmed that Scara5 expression decreased throughout the parenchyma. However, several positive Scara5 cells were found in the outer nuclear layer (arrowhead). D: During retinopathy, 2F8 positive cells were also observed in the outer nuclear layer (arrowhead), with a disposition compatible with that of Scara5 positive cells. 2F8 was revealed by DAB reaction and histological sections were counterstained with hematoxylin. GL, ganglion cell layer; INL, inner nuclear layer; ONL, outer nuclear layer. Scale bars: 45 µm (C); 20 µm (D).

Scara5 expression decreased approximately to a half during the establishment of retinopathy (Figures 10A and B). The immunolabeling with anti-Scara5 antibody confirmed that expression of this receptor decreased throughout the retina during retinopathy (Figure 10C). Several Scara5 positive cells were found within the outer nuclear layer (Figure 8C). The immunolabeling with anti-2F8 antibody, a macrophage marker [44], revealed the presence of 2F8 positive cells also within the outer nuclear layer, suggesting that the Scara5 positive cells observed may correspond to 2F8 positive macrophages that migrated to lesion sites [27].

### Scara5 was expressed in human retinas

Scara5 has been described in several organs and cell lines [45], but not in human retinas. To determine whether human retinas may contain Scara5, paraffin-embedded human retinal sections were analyzed by immunohistochemistry.

**Figure 11:**
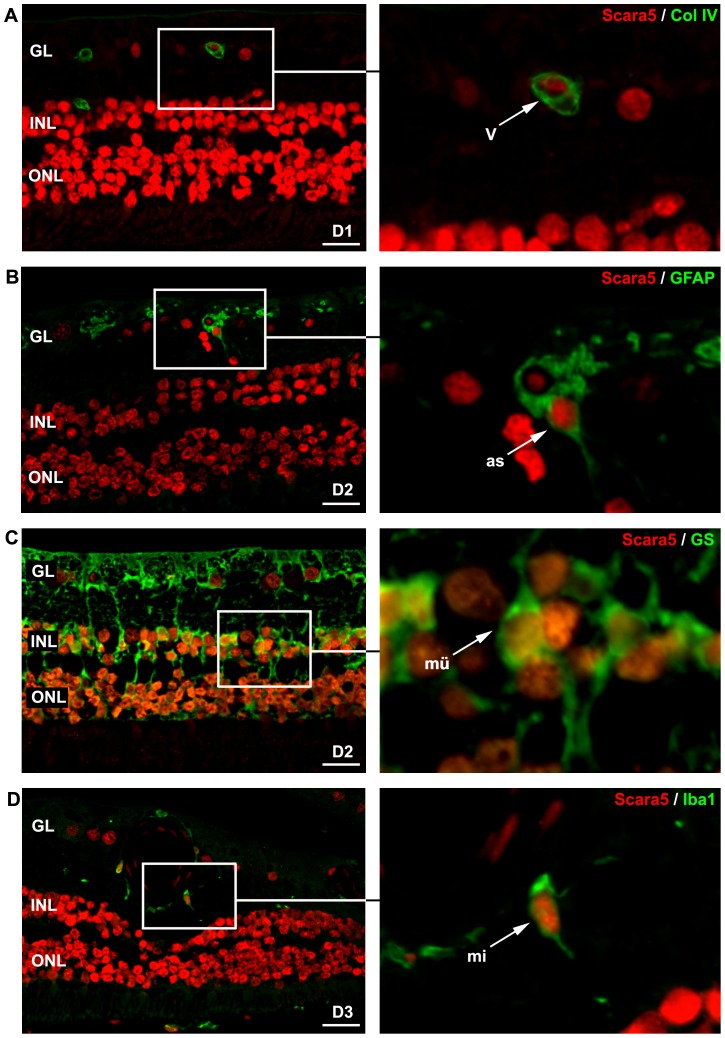
Scara5 was expressed in human retinal cells. Laser confocal analysis of double-stained paraffin-embedded human retinal sections with anti-Scara5 and anti-collagen IV, anti-GFAP, anti-GS and anti-Iba1 antibodies revealed that Scara5 was present throughout the retina, including endothelial cells, astroctyes, Müller cells and microglial cells, following the distribution pattern observed in mouse retinas. D1, D2 and D3, 42, 78 and 86-years-old healthy human donors, respectively; GL, ganglion cell layer; INL, inner nuclear layer; ONL, outer nuclear layer.: 22 µm (A); 21 µm (B); 24 µm (C); 28 µm (D).

Scara5 immunostaining was observed throughout the entire retina, at both cytoplasmic and nuclear levels, following the same distribution pattern observed in mouse retinas (Figure 11). Moreover, dual immunostaining for Scara5 and collagen IV, GFAP, GS and Iba1 confirmed Scara5 expression in vessel wall cells, astrocytes, Müller cells and microglia (Figure 11).

## Discussion

There is a general consensus that, in the retina, iron is obtained from the blood circulation through the classical transferrin endocytosis pathway at the level of the RPE and the inner BRB [4]–[6]. Recently, ferritin has been proposed as a new iron transport protein [19]. Ferritin can sequester 2000-fold more iron than transferrin [19], thus constituting a very efficient non-transferrin source of iron to tissues. Serum ferritin, mainly constituted by L-ferritin chains, is freely available in the bloodstream [46]. However, a ferritin pathway for iron delivery to the retina through L-ferritin binding to Scara5 has not been identified until now.

Our results showed that Scara5 was present in mouse and human retinas, throughout all the parenchyma layers, in different cell types, including endothelial cells. The retinal distribution of L-ferritin matched with that of its receptor Scara5. Furthermore, intravenously injected L-ferritin, in the form of HSF, crossed the inner BRB through its binding to Scara5 in endothelial cells, and thereafter reached the retinal parenchyma. Thus, suggesting the existence of a new pathway for iron delivery and trafficking in the retina.

Iron is required for the nuclear metabolism [47]. However, iron overload favors the production of reactive oxygen species. The hydroxyl radical, produced in the presence of the ferrous iron, is a powerful oxidizing agent who can promote mutagenesis, DNA strand breaks and activation of oncogenes [48]. Ferritin is present in the nucleus to prevent iron-induced oxidative damage [48]. In most tissues, nuclear ferritin is composed mainly by H-ferritin [48], [49], and some authors even deny the existence of L-ferritin in the nucleus [50]. However, our results demonstrated an important nuclear content of L-ferritin and its receptor Scara5 in retinal cells. Although H-ferritin also has a nuclear localization in retinal cells (data not shown), the presence of L-ferritin in the nuclei, with its specific iron nucleation function, should be of importance for counteracting the iron oxidative DNA damage.

Nuclear and cytoplasmic ferritins are the product of the same mRNA [50], but the precise mechanism for cytosolic ferritin translocation to the nucleus remains unclear [48]. The presence of nuclear Scara5 may represent a possible involvement in ferritin translocation to the nucleus.

In the murine model of photoreceptor degeneration induced by sodium iodate [29], Scara5 was downregulated. Thus, suggesting less influx of serum ferritin into the retina, and consequently a reduction in iron ligation. This decreased expression of Scara5 must be particularly harmful during iron acumulation conditions, such as diabetic retinopathy [11], where unliganded or incomplete liganded iron is associated with oxidative damage. This result points out Scara5 receptors as potential players implicated in retinopathy.

Scara5 is also downregulated in cancer, and the systemic upregulation of Scara5, through the treatment with Scara5 liposome complex, markedly inhibits tumor growth in mice [51]. This feature opens the possibility for using Scara5 as a potential therapeutic target to prevent free iron oxidative damage during retinopathy.
