# Correction: L-Ferritin Binding to Scara5: A New Iron Traffic Pathway Potentially Implicated in Retinopathy

**DOI:** 10.1371/journal.pone.0180288

**Published:** 2017-06-22

**Authors:** Luísa Mendes-Jorge, David Ramos, Andreia Valença, Mariana López-Luppo, Virgínia Maria Rico Pires, Joana Catita, Victor Nacher, Marc Navarro, Ana Carretero, Alfonso Rodriguez-Baeza, Jesús Ruberte

There is information missing from the Funding section. The correct funding information is as follows: This study was supported by grants from PTDC/SAU-ORG/110856/2009 from Fundação para a Ciência e a Tecnologia do Ministério da Educação e Ciência, Portugal; from Instituto de Salud Carlos III (PI12/00605), Ministerio de Ciencia e Innovacion, Spain; and from the Fondo Europeo de Desarrollo Regional (FEDER). The funders had no role in study design, data collection and analysis, decision to publish, or preparation of the manuscript.
